# Exploring the barriers to healthcare access among persons with disabilities: a qualitative study in rural Luuka district, Uganda

**DOI:** 10.1136/bmjopen-2024-086194

**Published:** 2024-11-02

**Authors:** Andrew Sentoogo Ssemata, Tracey Smythe, Slivesteri Sande, Abdmagidu Menya, Shaffa Hameed, Peter Waiswa, Femke Bannink, Hannah Kuper

**Affiliations:** 1 Disability Research Group, MRC/UVRI and LSHTM Uganda Research Unit, Entebbe, Wakiso, Uganda; 2 Department of Global Health and Development, London School of Hygiene & Tropical Medicine Faculty of Public Health and Policy, London, UK; 3 International Centre for Evidence in Disability, London School of Hygiene and Tropical Medicine, London, UK; 4 Division of Physiotherapy, Department of Health and Rehabilitation Sciences, Stellenbosch University, Stellenbosch, South Africa; 5 School of Public health, Makerere University College of Health Sciences, Kampala, Uganda; 6 Karolinska Institutet, Stockholm, Sweden

**Keywords:** Community-Based Participatory Research, Disabled Persons, Health Services Accessibility, Quality in health care, Patient-Centered Care, QUALITATIVE RESEARCH

## Abstract

**ABSTRACT:**

**Objective:**

The aim of the research was to explore the barriers to healthcare access for persons with various disabilities in rural Luuka district of Uganda. The findings will assist in appreciating the challenges persons with disabilities face in accessing Healthcare in a rural setting. These insights will contribute to the development of an intervention to improve healthcare access that is affordable, timely and acceptable.

**Design and participants:**

This qualitative study formed the exploratory formative phase of the ‘Missing Billion’ project. A total of 27 participants with disabilities—visual impairment (n=5), physical impairment (n=5), multiple impairments (n=6) and intellectual/ cognitive impairment (n=5) were purposively selected to participate in in-depth interviews conducted by two experienced researchers. Participants were identified through contact lists provided by the district disability focal person and local disability associations, with additional participants identified through snowball sampling. Interviews with persons with hearing impairment (n=6) were conducted by a researcher with hearing impairment. The interviews were audio/video recorded and transcribed to facilitate thematic data analysis. We used the disability-inclusive health ‘Missing Billion’ framework to map and inform the barriers.

**Setting:**

The study was conducted between September and November 2022 in rural communities in Luuka district, Eastern Uganda.

**Findings:**

On the demand side, challenges revolved around autonomy and awareness, limited access to health information, lack of financial capacity and dependence on caregivers for healthcare choices left persons with disabilities feeling disempowered. On the supply side, discrimination and negative attitudes from healthcare workers were reported as prevalent. Absence of healthcare workers and service delivery delays impacted on healthcare access, resulting in poor care. Inaccessible healthcare facilities compounded issues, as they had limited accessibility features.

**Conclusions:**

Complex and interconnected barriers underscore the pressing need for systemic changes to ensure equitable healthcare access for persons with disabilities in rural Uganda.

**STRENGTHS AND LIMITATIONS OF THIS STUDY**

- The participants of the study were sampled from a range of people living with a disability and representative of various disabilities to capture a diverse range of views and experiences.
- The depth of inquiry allowed us to identify potential areas of change for which solutions and interventions can be developed.
- Utilising an individual with lived experience of disability (hearing impairment) to collect data from participants with a hearing impairment helped bridge communication gaps and build trust, thereby enhancing the depth of the data collected.
- A limitation to this study was the very limited research conducted and initiatives for persons with disabilities in the region, which may have influenced the research participants to respond in a socially desirable manner.

## Introduction

 Access to healthcare is a fundamental human right that is recognised by various international and national legal frameworks.^1 2^ Despite this protection, people in low and middle-income countries (LMICs) face challenges in accessing quality primary healthcare.^3 4^ Poor access to healthcare may increase the risk of poor health outcomes and health disparities.^5^,^7^ Certain groups, such as the 1.3 billion persons with disabilities globally, are particularly disadvantaged.^8^ Failing to address the inclusion of persons with disabilities in the health system is, therefore, a violation of rights, and may also make it more difficult to achieve global health targets such as Sustainable Development Goal 3 and Universal Health Coverage.

Persons with disabilities frequently experience marginalisation, which manifests in multiple ways, such as discrimination, social stigma and physical barriers to participation in society.^9 10^ Additionally, persons with disabilities are more likely to live in poverty, due to limited access to education and employment opportunities.^8 10 11^ Inadequate education and economic instability perpetuates a cycle of poor health and poverty, restricting their ability to afford healthcare services and exacerbating their health conditions.^811^,^13^ They often experience additional barriers to accessing quality healthcare services, due to attitudinal/belief system barriers, informational barriers and practical and logistical barriers.^14^,^17^ Additionally, a recent systematic review demonstrated that attitudinal and belief system barriers, informational barriers and practical and logistical barriers greatly impact access to primary healthcare services for persons with disabilities in LMICs.^18^ These barriers deepen inequities in the quality of healthcare provided,^13^ and affect the full and equal enjoyment of all human rights and fundamental freedoms of persons with disabilities in line with the Convention on the Rights of Persons with Disabilities.^13 19^ These challenges are further compounded by the limited resources and infrastructure in LMICs, which often lack the necessary healthcare facilities, trained healthcare professionals and medical equipment to provide adequate healthcare services.^7 20^

Although barriers to healthcare for persons with disabilities have been studied in other settings, there is a lack of evidence on the lived experiences for persons with disabilities in rural Uganda. Yet, approximately 4.5 million Ugandans live with a disability with the greatest majority residing in rural areas, making them more vulnerable to healthcare access barriers.^21 22^ Rural access and utilisation of healthcare remain a challenge in Uganda due to the inequity in the health system, the interplay of poverty and healthcare access; compounded by the reliance on traditional, alternative and complementary medicines and care.^23^,^25^ Therefore, understanding the barriers to accessing healthcare and how these barriers affect persons with disabilities are important, as such knowledge can inform efforts to address these challenges.^18^ This study aimed to investigate the challenges that persons with disabilities experience in accessing healthcare services in Luuka district, Eastern Uganda. Uganda is a low-income country in Eastern Africa with an estimated population of 45.8 million in 2021 with less than 15% of the population living in urban settings.^26^ The country has a high burden of disease dominated by communicable diseases accounting for over 50% of morbidity and mortality, with rural poor communities being greatly affected.^27^ There are wide disparities in health status, along with major health system challenges, where health services are delivered through decentralised entities serviced by the public and private sectors.^27^

### Theoretical orientation

Access to healthcare is a complex issue, which has been conceptualised in diverse ways. We adopted the health systems framework conceptualisation developed and pilot tested by the Missing Billion Initiative.^28^ The framework describes the critical components of a strong disability-inclusive healthcare system. It proposes five dimensions of service delivery based on two arms of demand (autonomy and awareness, affordability) and supply (human resources, health facility, rehabilitation and assistive technology). We chose this framework because it is relevant for this context as it considers important objectives of disability-inclusive health systems that expect, accept and connect persons with disabilities to quality care critical for LMIC settings.^28 29^

## Methods

### Study design

This qualitative study formed the exploratory formative phase of the ‘Missing Billion’ project, aiming to explore healthcare access barriers among persons with disabilities. This project is generating evidence to inform community-based participatory approaches for improving healthcare access for persons with disabilities in Uganda.^30^

### Participants and setting

Primary data were collected from September to November 2022 from 27 purposively selected participants with varied impairments in Bukanga subcounty in rural Luuka district, Eastern Uganda. Luuka district comprises seven subcounties, one town council, and approximately 203 500 people. Participants were chosen to represent various ages, gender and self-identified impairment categories, including physical, hearing, visual, intellectual/cognitive and multiple impairments (table 1). Recruitment methods included obtaining contact lists from the district’s disability focal person, reaching out to local disability associations and utilising recommendations from previous participants (snowballing). From these contact lists, the researchers purposively selected participants who could represent the various categories of impairment, ages and gender for the study, ensuring a diverse and representative sample. Of the 28 individuals approached, only one declined participation.

**Table 1:** Participants’ profile

Participant number	Gender	Age range	Disability type
P-001	Female	35–45	Visual impairment
P-002	Male	50–55	Visual impairment
P-003	Male	60–65	Visual impairment
P-004	Male	35–40	Visual impairment
P-005	Female	60–65	Visual impairment
P-006	Female	35–40	Physical impairment
P-007	Male	50–55	Physical impairment
P-008	Female	25–30	Physical impairment
P-009	Male	30–35	Physical impairment
P-010	Female	40–45	Physical impairment
P-011	Female	30–35	Hearing impairment
P-012	Female	40–45	Hearing impairment
P-013	Female	20–25	Hearing impairment
P-014	Male	20–25	Hearing impairment
P-015	Male	25–30	Hearing impairment
P-016	Female	25–30	Hearing impairment
P-017	Male	30–35	Multiple impairments
P-018	Female	18–20	Multiple impairments
P-019	Male	20–25	Multiple impairments
P-020	Female	80–85	Multiple impairments
P-021	Female	25–30	Multiple impairments
P-022	Male	18–20	Intellectual/cognitive impairment
P-023	Male	20–25	Intellectual/cognitive impairment
P-024	Female	20–25	Intellectual/cognitive impairment
P-025	Female	20–25	Intellectual/cognitive impairment
P-026	Male	18–20	Intellectual/cognitive impairment
P-027	Female	25–30	Multiple impairments

### Data collection

Between September and November 2022, trained postgraduate social scientists (ASS and SS) with in-depth expertise in disability research and qualitative methods conducted individual in-depth interviews in English, Lusoga and Luganda (local dialects commonly used in the study area) to explore barriers to healthcare access among persons with disabilities in Luuka district, Uganda. The face-to-face interviews followed a semistructured guide (online supplemental appendix 1), developed by the research team based on related literature and gaps in knowledge. The guides were pilot-tested for clarity to ensure the questions were comprehensive and contextually relevant.

Topics included reasons for seeking care, support systems, challenges encountered and access facilitators. Interviews lasted 50–80 min, audio-recorded and supplemented with fieldnotes. A postgraduate research team member with a hearing impairment, a member of the hearing impairment community in Uganda and with expertise in qualitative research methodology conducted video-recorded interviews for participants with similar impairment, supported by a sign language interpreter. The rationale for video recording was to allow the sign language interpreter to voice over the videos, which were then transcribed for analysis. To ensure participant comfort and confidentiality, we followed strict protocols, including obtaining informed consent for the video recording and securely storing the video files.

Data collection occurred in private, participant-suggested locations including the participant’s home, community hall or health facility compound. The researchers (ASS and SS) compared notes and discussed emergent themes and ideas from the interviews during weekly debriefing meetings to ensure accurate data collection and interpretation, clarify any misunderstandings and generate to preliminary findings. A similar process was used in the analysis process. Saturation was reached when no new information emerged.

### Data management and analysis

Audio recordings of interviews were transcribed, with those conducted in Lusoga and Luganda, transcribed in the local dialect and later translated to English long with the fieldnotes by the research team (ASS and SS). The transcripts of the video recordings, which included the sign language interpreter’s voice-overs, were reviewed by SS who is fluent in both the local dialects and English. The accuracy of the translations was verified by AM (a sociologist), a native speaker of both local languages on the research team. The data management process was overseen by the first author (ASS), who has experience in disability research and qualitative methodologies, to ensure that the translations accurately reflected the participants' responses. The research team met regularly to discuss the data interpretation and develop the coding framework based on the Missing Billion framework.^28^

During the analysis phase, the researchers involved in coding and interpreting the data discussed their findings in regular team meetings with the entire research group. Summarised transcripts were indexed, then manually coded by two researchers (ASS and SS) using Microsoft Excel. Open coding identified emerging themes, while prominent excerpts were noted. Thematic data saturation was reached through analysis of all transcripts, ensuring exhaustion of new codes and themes. These preliminary themes were then refined and expanded throughout the data collection and analysis stages. Main themes were listed, with illustrative excerpts presented in the results. Thematic analysis, guided by a predetermined codebook based on initial research questions and emergent themes, explored perspectives on healthcare access barriers and enablers among persons with disabilities. Barriers were categorised using the Demand and Supply components of the Missing Billion Framework.^28^

### Ethics and informed consent details

The study obtained ethical approval from the Uganda Virus Research Institute Research Ethics Committee (REC ref GC/127/904) and the London School of Hygiene and Tropical Medicine (Ref 26715). Research clearance was granted by the Uganda National Council for Science and Technology (Ref SS1348ES) and the Luuka district local government.

Participants were provided with a study information sheet in their preferred language, along with a thorough verbal explanation of the study’s objectives, procedures and potential impact. This ensured that participants fully understood the significance of their involvement. It was emphasised that participation in the study was entirely voluntary, and participants were informed of their right to withdraw at any time without any consequences to their access to healthcare or other rights and privileges. Thereafter, participants were given opportunities to ask questions and voice concerns before agreeing to participate in the study and provide written consent. All participants provided written informed consent prior to any study-related activities.

### Patient and public involvement

Patients or the public were not involved in the design and conduct of this research.

### Rigour and trustworthiness

We employed a number of strategies to ensure rigour and trustworthiness in our study.

We considered maximum variation of the sample by deliberately seeking to include participants with a range of different impairments to capture a broad spectrum of experiences and perspectives. By including individuals with diverse types of disabilities, we aimed to obtain a more comprehensive understanding of the barriers faced by various subgroups within the population. This approach ensures that the findings reflect the diverse needs and challenges experienced by people with disabilities, rather than focusing on a homogeneous group that might not represent the broader population. The face-to-face in-depth interviews were majorly conducted in convenient and private participant-suggested locations including the participant’s home, community hall or health facility compound to ensure balance of power during the interviews. Additionally, interviews with persons with hearing impairment were conducted by a research team member who has a hearing impairment with support of a sign language interpreter.

The analytical process was conducted independently by two researchers (ASS and SS) who have extensive experience in disability research and qualitative research methodologies. Data from the interview and fieldnotes were triangulated, also to ensure that the results were confirmable. Additionally, the research team had multiple debriefing sessions to discuss emerging themes and ensure a consistent and comprehensive interpretation of the data. At these session meetings, we critically evaluated our biases and assumptions throughout various study stages. The team members involved in the study have expertise in disability research, participatory community-based research, health systems and qualitative research methodology. We analysed and presented the findings of the study based on participants’ experiences and the findings presented in the Results section represent a significant amount of the study results reflected in the representative excerpts. The consolidated criteria for reporting qualitative research checklist^31^ were used to aid reporting this study.

## Results

The demographic characteristics of the 27 persons with disabilities interviewed are shown in table 2.

**Table 2:** Participant demographics of interviewed person with disabilities (N=27)

Category	Characteristic	n (%)
Gender	Female	15 (56)
	Male	12 (44)
Impairment	Hearing	6 (22.25)
	Physical	5 (18.5)
	Visual	5 (18.5)
	Intellectual/cognitive	5 (18.5)
	Multiple	6 (22.25)
Age	Median age (IQR)	31 (21–50) years
Occupation	Formal employment	8 (30)
	Informal employment	15 (56)

**Figure 1:**
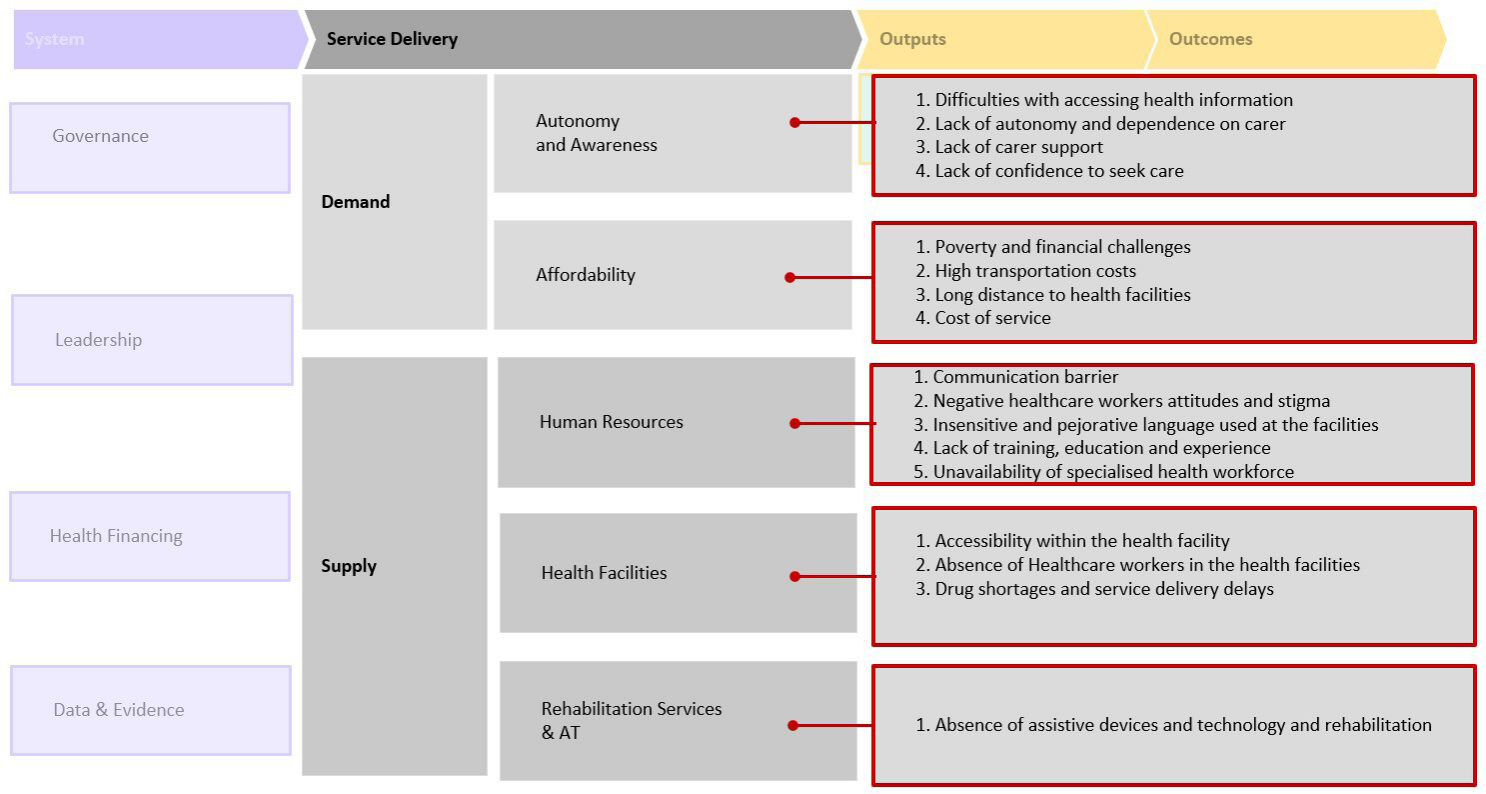
Barriers to access to healthcare based on the health systems framework proposed by the Missing Billion initiative.^28^ AT, assistive technology.

Barriers related to access to healthcare service were categorised into Demand (Autonomy and Awareness, affordability) and Supply (Human Resources, health facilities, rehabilitation services and assistive technology) service delivery components as shown in figure 1.

### Demand side barriers

#### Autonomy and awareness

##### Difficulties with accessing health information

Participants described challenges accessing health information, impacting their decision-making regarding seeking care. Typically disseminated through media channels such as radios, televisions and newspapers, health information may be less accessible to certain categories of persons with disabilities, hindering their ability to know why, where, when and how to visit a health facility.

*Health information is not tailored. One time they announced on the local radio that people needed to go for COVID-19 vaccination but for us the deaf, we did not go to the hospital because we were unaware.* (P-015, hearing impairment)

Low levels of literacy and the lack of accessible formats make it difficult for some persons with disabilities to comprehend information, education and communication materials such as fliers, leaflets, audio-visual screens and brochures at health facilities. In the words of a participant:

*Information is given in standard formats and since many of us never went to school, we don’t receive this information or understand it. There are no picture or braille formats.* (P-010, physical impairment)

### Limited decision-making and dependence on carer

Accessing healthcare services for persons with disabilities frequently depended on caregiver or family member support. Participants universally reported limited opportunities for independent decision-making regarding how, where and when to access healthcare services. For instance, one participant shared their experience, stating:

*When I want to go to the facility, I need a person to guide me there. When am given medicine, I need someone to explain it to me and hand it to me. I am blind and cannot do much, so I depend on others even for healthcare. I am like a little child*. (P-020, Multiple impairment).

Another participant narrated that:

I am not the one who decides if I need to go to hospital or where to go because of my disability, I just sit there. Also, because am not the one who pays. At the moment, I lack someone to take care of me and to pay for my medical treatment. My daughter who used to do all these things just passed on a few months back. (P-022, cognitive impairment)

Participants reported limited direct communication with healthcare workers, who prioritise interacting with caregivers over persons with disabilities, reducing their willingness to revisit the clinic. For example, one participant described relying heavily on a caregiver to access antenatal services, highlighting reduced autonomy.

*When I was pregnant with my first child, I went to the hospital. When the doctor discovered I was deaf, he left and never wanted to help me out. I was helpless. My mother later came and helped facilitate communication between me and the doctor. How do you expect me to go back to the health facility.* (P-016, hearing impairment)

Participants also identified the absence of caregivers as a challenge in accessing healthcare services. Additionally, due to poverty, persons with disabilities often depend on family members and friends for financial support when seeking healthcare. One participant noted:

*I need help and support in walking to the health facility where I can get the medication. If there is no one, I cannot go and many times because they are paying for you, you have no choice on where they take you.* (P-002, Visual impairment)

Some participants were deterred from seeking assistance due to the negative attitude and perceptions of caregivers who viewed them as a ‘burden’. These challenges are highlighted in the following quote:

*I am not able to reach the health facility unless there is someone willing to take me. I always have to beg someone who doesn’t think I am a problem to them; to take me to the hospital. Am helpless and the little boy who could take me went back to school. If I am not supported, I am not able to go to hospital*. (P-020, Multiple impairment)

#### Lack of confidence to seek care

Some participants hesitated to seek care due to anticipated fear, embarrassment and self-stigma. Concerns about others’ perceptions, especially regarding sexual reproductive health services, compounded this hesitation. These concerns are illustrated in the following excerpts.

*We are afraid of crawling from home, thinking - ‘how will they see me crawling to the health centre or getting sexual and reproductive health services when I reach the facility!’ We have social phobia and are frightened to move even when we are ill and need medical assistance. This is because someone has been laughed at previously and feel they will be laughed at then again due to their disability.* (P-006, physical impairment)

Additionally, discrimination, poor health worker attitudes and service delivery delays were reported, further deterring participants from seeking care.

*I had malaria and went to the hospital. I wrote a note to the nurse that I was sick and a deaf patient. Later, she started complaining that she had called out my name to go for the malaria test and I did not respond. She chased me away in public. I felt ashamed and disappointed. I have never gone back.* (P-016, hearing impairment)

Long waiting times was a significant barrier, further deterring participants from seeking necessary care and losing confidence in the healthcare system. These delays were not just a matter of inconvenience but were deeply intertwined with the systemic challenges facing rural healthcare facilities. As one participant poignantly described,

*We have no trust and confidence in the system. Imagine you had a bad night so you arrive at the health centre at 8am and sit for long at the triage in pain anticipating that they will come. On a lucky day they appear at 11am, then they delay attending to you but after waiting for so long and at the end they just give you Panadol.* (P-022, intellectual impairment)

### Affordability

#### Poverty and financial challenges

Participants also identified financial constraints as a barrier to accessing healthcare, especially as many participants were unemployed. As one participant noted:

*To get a health service, you must have money. If you don’t have the money, no health worker can give you the treatment and many persons with disabilities struggle financially.* (P-009, physical impairment)

Costs incurred included consultation fees, and purchasing medications during drug stockouts, yet exacerbating financial strain for those with unstable incomes. Many resorted to private purchases, further increasing healthcare expenses. In some cases, individuals had to pay bribes to see a healthcare worker or receive additional support. Reflecting on the challenges faced, a participant highlighted:

*I was told there are no drugs, so I needed to go and buy, but I had no money. So, I returned home, sold off some bananas in the garden to buy some of the medicines worth 5000 shillings. I negotiated and paid the healthcare worker near my home to administer the injections because I had no money to take me back to the health facility. By the time I sold the other bananas to buy more medicines to complete the prescription, the surgeon said it was too late, I needed surgery that would cost me 300 000 shillings, which I have failed to get to date.* (P-004, visual impairment)

Transport costs posed a significant challenge for persons with disabilities, particularly due to reliance on costly public transportation like boda-bodas (motorbike taxi) and matatus (shared taxis). Participants often incurred double costs, covering both their own transportation and that of their caregivers. Wheelchair users faced additional expenses, as they had to pay for the wheelchair. Reflecting on these challenges, a participant emphasised:

*Transport to the health facilities is a big challenge because a boda-boda to the health facility, costs 3500–5000 shillings one way. The boda-boda or taxi people will ask if they are taking you plus the wheelchair. When you tell them the wheelchair are my legs, they respond that you have to pay transport fee for the wheelchair too. You wonder, ‘why I am supposed to pay for my legs [wheelchair]!’ If you cannot pay, they leave you by the roadside.* (P-019, multiple impairments)

Linked to transport cost challenges, the long distances to health facilities emerged as another significant hurdle. Persons with disabilities frequently had to travel several kilometres to reach the nearest government health facilities. Private health facilities in their communities were expensive and lacked services tailored to their needs.

*I struggle moving yet the health facilities are extremely far. I must confess, it is very hard, and I have taken a long time, minus going to a health facility because the nearest is around 8 km from here which is very far when you need to see a healthcare worker or receive treatment.* (P-002, visual impairment)

As a result, persons with disabilities often delayed seeking care until their conditions worsened, missed appointments or did not visit health facilities at all. This posed a significant risk for those with comorbidities such as hypertension or diabetes as routine visits were missed potentially leading to loss of care.

*The health facilities are very far from us and reaching them becomes very hard and costly when you have to go there regularly for check-up and treatment. You imagine because of my diabetes I need to go to the health centre more often, but I have missed for the last 5 months.* (P-017, Multiple impairments)

Many persons with disabilities interviewed stated that they cannot afford specialised care due to inflated costs involved and most referral/specialised care services are accessed in other districts. Because of this issue, persons with disabilities take incomplete doses of medications, miss appointments, delay to seek healthcare services or do not get treatment at all as expressed in the excerpt:

*At our health centre, it is impossible to get all the treatment. They give you some and tell you to buy the rest outside the hospital pharmacy. Sometimes you are referred for specialist care which is extremely expensive and challenging for us to afford as some of us are poor and have a lot of challenges.* (P-015, hearing impairment)

### Supply side barriers

#### Human resources

##### Negative attitudes and discrimination from healthcare workers

Participants experienced negative attitudes and discrimination by some healthcare workers at the health facilities and in the communities. Negative health worker’s attitude can be manifested as, offensive, belittling, undignified and irrational language, and unkind names (related to their disabilities). For instance, one participant described how they are often labelled, described, and addressed by their disability.

*I found a doctor speaking English thinking that I cannot understand. He commented, ‘I don't want to touch women with disabilities because they are dirty. I kept quiet and he insisted, I won't examine her. If it weren’t for the nurse convincing him that I was clean, I wouldn't be helped.* (P-001, visual impairment)

Healthcare workers were reported to hold inappropriate assumptions about persons with disabilities, such as questioning their ability to become pregnant and perceiving them as time-consuming, difficult to manage and increase burnout as they require the health worker’s support throughout the health-seeking process. These negative attitudes contribute to stigma, impacting healthcare access, service quality and health-seeking behaviours of persons with disabilities.

*There is the stigma, discrimination and ‘bad vibe’ at the health centre, like when a lame person comes to the facility, the healthcare workers often start asking, ‘why did you even bother to come to waste our time, all because you cannot act very fast.* (P-010, physical impairment)

Participants recounted negative attitudes abusive, discriminative and dehumanising language used by health workers during antenatal or maternity care. For example, health workers wonder why and how women with disabilities get pregnant and think they cannot deliver normally (without caesarean section).

*The healthcare workers are rude with persons with disabilities asking them why they get pregnant to disturb the healthcare workers. The nurse asked me to prove that I was pregnant and how I got pregnant. This angered me a lot.* (P-008, physical impairment)

### Lack of knowledge and skills around disability

Participants mentioned that the healthcare workers lack the knowledge and skills of how to manage and deal with persons with disabilities. They noted that many times the healthcare workers managed and attended to them with little or no consideration and reasonable accommodation. Participants also shared that healthcare workers frequently focus on the impairment/disability rather than the presenting problem (ie, diagnostic over-shadowing).

*It is just in the mind of health workers to think that persons with disabilities are not easy to serve because they lack the knowledge, skills, experience, and training in caring for them. They immediately think you have come for eye treatment even when you have other ailments*. (P-002, visual impairment)

Healthcare workers were reported to give insufficient time to carefully listen to the needs of persons with disabilities, potentially due to insufficient time set aside for medical examination and procedures and long lines outside the consultation rooms. Yet persons with disabilities often travelled miles to seek for this service, which demoralises them to return for the subsequent visits.

*… when they [healthcare workers] see someone with a disability, they see such patients as a problem or a challenge that will burden them. They will not give you enough time to share your problem. So, you don’t get the care, and you cannot come back again.* (P-006, physical impairment)

Linked to accessing health information, participants noted that poor physician–patient communication may limit persons with disabilities from engaging with the healthcare workers, receiving relevant health information, making proper consultations about their health and consequently received the wrong diagnosis or inappropriate medication.

### Health facilities

#### Access challenges within the facility

Participants pointed out that many structures at health facilities are inaccessible; lack access ramps, rails and clear signage. Wheelchair users reported that they must get off the wheelchairs to be attended to. The facilities were reported to lack sign language interpreters or guides to support persons with disabilities.

*I feel frustrated that even if I get to the health facility, am going to be outside because no one will push my wheelchair into the building without the ramps. When I am well dressed, I have to crawl on the floor from one place to another and get dirty in order to get treatment. That alone hinders me from going to the hospital*. (P-009, physical impairment)

Lack of accessibility is not only relevant to the entrance to the facility but also equipment and internal structures.

*Most of the health facilities are not disability friendly. The toilets are not convenient, persons with disabilities will come to the health facility to get medical attention and want to ease themselves, get a urine, or stool sample and you find that the lavatories are not user-friendly* (P-012, hearing impairment)

Lack of adjustable beds to support the access and mobility for persons with disabilities is another issue. As one woman describes:

*You are pregnant, but the healthcare workers are telling you without any sympathy to get onto the bed that is raised high that even a person without disabilities could find a challenge to climb. They will tell you that if you were able to get onto the bed with your man while getting pregnant, why do you find it hard to get onto the delivery bed - very rude*. (P-006, physical impairment)

### Service delivery delays

Participants highlighted significant difficulties in navigating the healthcare system, exacerbated by service delivery delays. Uncertainty about where to start and who to contact on reaching the facility was a common challenge. Moreover, participants noted the absence of special service delivery arrangements for persons with disabilities, who were required to stand in long queues despite their unique challenges.

*Persons with disabilities take long to receive care. I went there early morning at about 6:00am but they first work on people without disabilities and thereafter serve those with disabilities. You wait long time to get served and spend the entire day lined up in the sun. I felt angry and discriminated.* (P-027, multiple impairment)

*…. Imagine you had a bad night so you arrive at the health centre at 8am and sit for long at the triage in pain anticipating that they will come. On a lucky day they appear at 11am, then they delay attending to you but after waiting for so long…* (P-022, intellectual impairment).

### Rehabilitation services and assistive technology

#### Absence of assistive devices, technology and rehabilitation

Participants noted a major challenge in accessing healthcare due to the absence or unaffordability of appropriate assistive devices like wheelchairs, white canes, hearing aids and sunglasses even at the facilities. This resulted in mobility challenges, with many relying on assistance from well-wishers or non-governmental organisations for these devices.

*I cannot travel to the health centre anymore on this dusty road because I can no longer see clearly as my goggles which [mentions name of charity] gave me broke when I fell. They were protecting me from the direct light and dust that cause a lot of pain. Buying new goggles is costly.’* (P-001, visual impairment)

Participants mentioned that the health facilities also lacked wheelchairs and adjustable beds and other assistive technology to support persons with disabilities within the facilities. They equally indicated the absence of close-by rehabilitation services.

*…that’s where the problem is because most health centres don’t have assistive devices like wheelchairs, hearing and visual aids. Therefore, striving to reach the facility and you cannot be helped appropriately is not beneficial* (P-010, Physical impairment)

*… you go to the health facility and the doctor says you need physiotherapy sessions or a therapist for speech to help you, but these services are not even here in our district let alone the doctor not knowing where exactly to refer you to. You go as far as Iganga or Jinja and these services are not there.* (P-024, intellectual impairment)

### Lack of specialised health workforce

Healthcare workers were reported to lack specialised training required to treat persons with disabilities and manage certain conditions related to disability. Participants reported that healthcare workers only dealt with their general healthcare needs and those requiring specialised care were referred to high-level health facilities outside the district, which were difficult and expensive to access. Participants also noted a lack of social workers and rehabilitation health professionals, in all the health facilities in the district, to address the psychosocial and rehabilitation needs of persons with disabilities.

*We have health needs that need certain experts, and we do not have these at the facilities. The doctor will only give you medicine to relieve the symptoms and tell you to go and look for a specialist, who many times is expensive, and you have to travel to Kampala to see them.* (P-027, multiple impairment)

## Discussion

Our study highlights healthcare inequities faced by rural Ugandans with disabilities. On the demand side, communication barriers such as the lack of accessible formats like sign language interpretation, braille or simplified language made it difficult for individuals to fully understand their health conditions and the available healthcare services. The limited awareness of available services hindered informed decision-making to seek healthcare leading to underutilisation of available health services. Inaccessible media and low health literacy exacerbated these challenges. Additionally, reliance on caregivers for assistance, coupled with a lack of direct communication with healthcare professionals, often resulted in disempowerment as the persons with disabilities frequently felt excluded from conversations about their own health, leading to a loss of autonomy and confidence in making healthcare decisions. Financial obstacles were another significant barrier, as many individuals with disabilities in rural Uganda live in poverty and cannot afford the costs associated with healthcare, including transportation to facilities, medication or specialised care. This financial strain was often exacerbated by self-stigmatisation, and low self-esteem, which deterred some from seeking care due to fears of discrimination, feeling like a burden.

On the supply side, we found discriminatory behaviours and negative attitudes exhibited by healthcare providers. These attitudes often stem from a lack of understanding and awareness about the needs and rights of persons with disabilities. Participants denoted significant deficiencies in training and experience of healthcare workers pertinent to serving persons with disabilities. Additionally, service delivery delays, a notable absence of essential assistive devices and technological aids crucial for accommodating persons with disabilities complicated access to healthcare. The inaccessibility of healthcare facilities significantly exacerbated these challenges, with these establishments lacking requisite features such as ramps, handrails or accessible toilets to adequately accommodate persons with disabilities.

The present study found that multifaceted challenges such as lack of confidence to seek care, poor carer and health worker attitude created a sense of stigma and dejection to accessing healthcare similar to previous studies.^9 13 18^ The lack of confidence to seek care was often rooted in past negative experiences with the healthcare system and medical mistrust common in rural communities. This is exacerbated by experiences with discrimination and marginalisation similar to findings of other studies.^10 32^ This lack of confidence to seek care is often compounded by poor attitudes of healthcare workers, negative interactions with healthcare providers and self-stigmatisation among persons with disabilities contributing to healthcare access and outcome disparities.^8 13 33^ For instance, participants reported feeling judged or treated with condescension by healthcare workers, leading to feelings of shame and a reluctance to seek help.

The barriers to access to healthcare identified in our study are consistent with qualitative studies undertaken in other African countries. For example, poor health literacy, lack of finance and self-stigma, compounded by poor capacity of health workers to treat persons with disabilities, discrimination and inadequate health facilities were highlighted in Zimbabwe.^34^ In South Africa, living with a disability in a rural setting was linked to discrimination, social exclusion, isolation and difficulties accessing services, with context-specific factors such as mortality rates, recurring violence and government policy issues playing significant roles in shaping this experience.^35^

Our findings align with a study conducted in Malawi that found that persons with disabilities faced multiple, complex barriers to accessing healthcare and identified three key barriers—cost, insufficient healthcare resources and dependence on others.^29^

Additionally, Hanass-Hancock *et al*^36^ revealed a multifaceted perspective on disability-related costs, encompassing support for survival and safety, service accessibility and community participation, with variations in experiences dependent on care requirements, accessibility factors, service availability and assistive device knowledge.

The intersectionality and interconnectedness of these challenges underscore the importance of a comprehensive approach to addressing healthcare inequities, one that considers the full spectrum of barriers that persons with disabilities face in accessing healthcare. The health access barriers identified underscore the urgent need for policy and infrastructure reforms that prioritise accessibility and inclusivity in healthcare settings. For example; healthcare workers must adopt a patient-centred approach, involving persons with disabilities in the decision-making and care processes throughout the consultation, examination and referral stages.^28^ Training programmes should include evidence-based modules on disability and inclusive healthcare, developed and delivered in collaboration with persons with disabilities.^37^ Additionally, developing and availing Continuing Professional Development courses and Continuous Medical Education sessions on disability inclusion as part of these programmes can ensure that healthcare providers remain updated on best practices and are continuously improving their skills in inclusive healthcare. Evaluation of gaps and training needs in the existing medical education curriculum in Uganda is crucial.^37^,^39^

Policymakers can address affordability of healthcare challenges by creating employment opportunities and incentivising employment for persons with disabilities, tailoring ongoing poverty alleviation and social protection mechanisms^36^ similar to what has been done in Uganda’s education sector.^40^ Health insurance has been reported to reduce out-of-pocket expenditures and improve access to healthcare for persons in need of life-long medical care access in Uganda and warrants further exploration.^41^ Provisions and prioritisation for persons with disabilities in national health insurance schemes, focusing on equity and need, is vital to reduce out-of-pocket expenses to reduce economic vulnerability.^36 42 43^

Infrastructure modifications in healthcare facilities, such as ramps, hand rails, adjustable examination tables and accessible toilets, and surrounding environments to accommodate persons with disabilities can enhance accessibility and their overall healthcare experiences.^28^ Making assistive technology, rehabilitation and specialist services available and affordable is essential for inclusion, independence and active participation in the health seeking and care journey.^44^ Additionally, healthcare workers should develop appropriate referral pathways for rehabilitation and specialist services to offer additional care and support for persons with disabilities.^28^

### Strength and limitations

The utilisation of qualitative methodology unpacks persons with disabilities experiences in accessing healthcare and barriers faced from their perspectives and in their own words. Another strength of this study lies in the diversity of our sample, which encompassed a wide range of persons with disabilities. The disability inclusivity process of using a researcher with a hearing impairment to collect data from persons with hearing impairment enhanced the authenticity and depth of the data collected. Additionally, the depth of inquiry allowed to identify potential areas of change for which solutions and interventions can be developed. The analysis was based on the Missing billion inclusive health systems framework^28^ that illustrates how systemic factors influence healthcare service delivery, relevant to persons with disabilities.

This study has some limitations. Given that we specifically interviewed participants from a rural setting, it is possible that barriers experiences may differ from those of individuals in urban or other settings. We focused only on access to primary healthcare. Thus, results cannot be generalised to specialist healthcare or to care seeking in urban regions. We also note that very limited research and initiatives for persons with disabilities have been conducted in the region which may have influenced the research participants to respond in a socially desirable manner. Additionally, we did not conduct repeated interviews or provide participants with their transcripts for feedback. Future studies may need to consider involving healthcare workers and members involved in the healthcare system who may have alternative perspectives.

## Conclusion

The multifaceted and interconnected barriers experienced by persons with disabilities accessing healthcare in rural Uganda underscores the pressing need for systemic changes to ensure equitable health access. Interventions should be co-created with persons with disabilities to ensure they are contextually relevant and address the unique challenges faced by this population. Key strategies might include training healthcare providers on disability-inclusive practices, improving the physical accessibility of health facilities, and strengthening community-based support systems. Additionally, policymakers should prioritise the inclusion of persons with disabilities in health policy development and resource allocation to ensure that their needs are adequately met. Collaboration between government agencies, non-governmental organisations and local communities will be essential to implement these changes effectively and sustainably.

## supplementary material

10.1136/bmjopen-2024-086194 online supplemental file 1

10.1136/bmjopen-2024-086194 online supplemental file 2

## Data Availability

All data relevant to the study are included in the article or uploaded as supplementary information.
