# Supplementary material for: Exploring the barriers to healthcare access among persons with disabilities: a qualitative study in rural Luuka district, Uganda
Source: BMJ Open. 2024 Nov 2;14(11):e086194. doi: 10.1136/bmjopen-2024-086194 (PMC11535691; doi:10.1136/bmjopen-2024-086194)
Supplement: online supplemental file 2 [file bmjopen-14-11-s002.docx]

**Table 1 : Participants’ profile**

| **Participant No.** | **Gender** | **Age** | **Disability type** |
| --- | --- | --- | --- |
| P-001 | Female | 43 | Visual Impairment |
| P-002 | Male | 50 | Visual Impairment |
| P-003 | Male | 62 | Visual Impairment |
| P-004 | Male | 40 | Visual Impairment |
| P-005 | Female | 60 | Visual Impairment |
| P-006 | Female | 45 | Physical Impairment |
| P-007 | Male | 50 | Physical Impairment |
| P-008 | Female | 20 | Physical Impairment |
| P-009 | Male | 34 | Physical Impairment |
| P-010 | Female | 40 | Physical Impairment |
| P-011 | Female | 30 | Hearing Impairment |
| P-012 | Female | 44 | Hearing Impairment |
| P-013 | Female | 21 | Hearing Impairment |
| P-014 | Male | 21 | Hearing Impairment |
| P-015 | Male | 37 | Hearing Impairment |
| P-016 | Female | 26 | Hearing Impairment |
| P-017 | Male | 30 | Multiple Impairments |
| P-018 | Female | 18 | Multiple Impairments |
| P-019 | Male | 23 | Multiple Impairments |
| P-020 | Female | 80 | Multiple Impairments |
| P-021 | Female | 25 | Multiple Impairments |
| P-022 | Male | 18 | Intellectual/ Cognitive Impairment |
| P-023 | Male | 23 | Intellectual/ Cognitive Impairment |
| P-024 | Female | 22 | Intellectual/ Cognitive Impairment |
| P-025 | Female | 20 | Intellectual/ Cognitive Impairment |
| P-026 | Male | 19 | Intellectual/ Cognitive Impairment |
| P-027 | Female | 25 | Multiple Impairment |
