# Supplementary material for: Exploring the barriers to healthcare access among persons with disabilities: a qualitative study in rural Luuka district, Uganda
Source: BMJ Open. 2024 Nov 2;14(11):e086194. doi: 10.1136/bmjopen-2024-086194 (PMC11535691; doi:10.1136/bmjopen-2024-086194)
Supplement: Supplementary file 3 [file bmjopen-2024-086194.reviewer_comments.pdf]

PEER REVIEW HISTORY

BMJ Open publishes all reviews undertaken for accepted manuscripts. Reviewers are asked to complete a checklist review form and are provided with free text boxes to elaborate on their assessment. These free text comments are reproduced below.

ARTICLE DETAILS

Title (Provisional)

Exploring the barriers to healthcare access among persons with disabilities: a qualitative study in rural Luuka district, Uganda.

Authors

SSEMATA, ANDREW SENTOOGO; Smythe, Tracey; Sande, Slivesteri; Menya, Abdmagidu; Hameed, Shaffa; Waiswa, Peter; Bannink, Femke; Kuper, Hannah

---

VERSION 1 - REVIEW

Reviewer 1

Name Kumurenzi, Anne

Affiliation University of Rwanda College of Medicine and Health Sciences, Physiotherapy

Date 07-Jul-2024

COI None

This paper provides additional information on the barriers to accessing healthcare services, specifically in remote and underserved areas, such as rural regions. However, the strategies to mitigate these barriers need to be explicitly articulated. This information is paramount, as it will help any stakeholder interested in supporting this population devise practical approaches.

• The quotes from participants need to be reviewed, and suggestions are included in the comments in the paper.

• The results sections need more clarity, as some sections do not include participants' quotes, only the authors' comments on the issue.

• It is unclear where the interview questions were retrieved from. Were they from the literature or self-developed?

• The discussion needs to be expanded as there are many results to discuss.

• Including the interview guide could provide readers with additional information about the collected data.

• Update some of your references

• Refer to the paper for additional comments.

****The reviewer provided a marked copy with additional comments. Please contact the publisher for full details.**

Reviewer **2**

Name **Masuku, Khetsiwe P**

Affiliation **University of the Witwatersrand Johannesburg, Speech Language Pathology**

Date **19-Jul-2024**

COI **I have no competing interests**

Thank you so much for awarding me the opportunity to review this manuscript. I do believe that the manuscript presents a pertinent issue, especially in the African context. While I do believe that the manuscript is publication worthy, I don't believe that the following should be addressed before it can be considered for publication:

Abstract:

1. I would suggest adding a sampling strategy in the abstract

2. line 51, correct the typing error. There are two full stops.

Article summary under strengths and limitations:

3 .Maybe a another strength is the use of a persons with disability to collect data from other persons with disabilities, in this case persons who are deaf.

Introduction:

4. Line 93 remove double full stops.

5. Line 107-108, maybe a brief explanation of why rural Ugandan contexts are more susceptible to these barriers.

Methods:

6. Line 134-135, check the grammar in sentence 135 " This project generating evidence generates evidence to inform......"

7. Participants and settings-More clarity is required regarding recruitment. What was done with the lists received from the district focal person? How did you curb form cohesion in situations where the researcher contacted the participants directly and not the participants contacting the researchers?

8. Data Collection-More details regarding the persons who conducted the study, their qualification, training on the research methodology. Training of the person who conducted the interviews with deaf participants. etc.

9. Data Collection line 157-158-Did anyone check the accuracy of the voice overs? If so, who?

10. Data Collection- How were differences treated in data interpretation?

11. Data management and analysis-Who did the translations from Lusoga and Luganda? When translations were done, how was it ensured that no meaning was lost?

12.How was trustworthiness, the equivalent of validity and reliability in qualitative research ensured?

13. Results- In the argument for the study (introduction section), reference was made to the rural context of Uganda compounding access to healthcare. Even in the discussion similar reference is made using Neil and Penn, 2015. How many of these participants were from rural and how many were from urban areas? This information needs to be mentioned.

14. Findings-I would suggest that in the presentation of the results the tenets of the framework are specified and so should the themes and sub-themes for ease of reference. The way in which the results are currently presented requires the reader to deduce this information.

15. Findings 205-206-This makes it seem like these are the main methods of health information sharing, however then in lines 213-216, it seems like there are more avenues for information sharing within the healthcare facilities. Please make sure that there are no contradictions.

16. Findings-The results are interesting but limited to the themes. The sections on the different themes present explanations related to the theme. The themes encompass a lot of elements (sub themes), but one excerpt pertaining to only one aspect of the different elements presented in the one theme. Better to present an excerpt about a sub-theme in a paragraph as opposed to the theme. For example, in the theme of lack of confidence to seek care, there is a lot to unpack, which is bundled as "lack of confidence". Key issues which should be stand alone sub-themes e.g stigma should be teased out further, HCP attitudes, service delivery etc.

17. Discussion- The discussion section is very wanting.

18. References-It is very concerning that dated references, some as far back as 2013 have been used when there is current literature on access to healthcare in Sub Saharan African Countries from 2019- currently. Can this be addressed please

19. Editorial care- I would recommend that this manuscript undergoes editing.

VERSION 1 - AUTHOR RESPONSE

15th August 2024.

To: Nicole Martin

Research Editor

BMJ Open

Dear Editor,

We are resubmitting a research article titled " “Our health facilities are not disability friendly.” Barriers to healthcare access among persons with disabilities in Uganda: a qualitative study." - bmjopen-2024-086194 – whose new title based on your comments now reads “Exploring the barriers to healthcare access among persons with disabilities: a qualitative study in rural Luuka district, Uganda.” for consideration for publication in BMJ Open with point-by-point response to the reviewers’ comments as below:

Reviewer 1:

This paper provides additional information on the barriers to accessing healthcare services, specifically in remote and underserved areas, such as rural regions. This information is paramount, as it will help any stakeholder interested in supporting this population devise practical approaches. The quotes from participants need to be reviewed, and suggestions are included in the comments in the paper.

Response: Thank you for the helpful suggestions. We have revised the results of our manuscript accordingly and included additional excerpts.

• The results sections need more clarity, as some sections do not include participants’ quotes, only the authors’ comments on the issue.

Response: Thank you for highlighting this issue. We have revised the results section and added participant excerpts. We acknowledge that including participant excerpts is essential for providing clarity and supporting the findings presented in the results section. Although we were unable to include as many quotes as we would have liked, we have included representative direct participant excerpts that best illustrate the key themes, ensuring that

the narrative around the findings is as clear and detailed as possible, reflecting the perspectives shared by participants during the study.

• It is unclear where the interview questions were retrieved from. Were they from the literature or self-developed?

Response: Thank you for your comment. The interview questions were developed by the research team based on the review of relevant literature and gaps in knowledge. The guides were pilot tested for clarity and relevance. We have clarified this in the manuscript. (see lines 179-181)

• Including the interview guide could provide readers with additional information about the collected data.

Response: Thank you for the suggestion. We agree that including the interview guide would provide readers with valuable context and a better understanding of the data collection process. We apologize for the oversight of not including Appendix 1 during the initial submission, which contains the interview guide.

• Update some of your references

Response: Thank you for the feedback. We have reviewed the references and identified those that require updating. We have included some more recent and relevant sources to ensure that the manuscript reflects the latest research and developments in the field.

• Refer to the paper for additional comments.

Response to additional Comments inserted in the manuscript

Is this the next part of the project? Otherwise, a single intervention cannot address the issues related to barriers to healthcare access.

Response: Yes indeed, the findings from this manuscript have been used to inform the development of the PLA-D intervention to improve healthcare access for persons with disabilities

Were some interviews audio and other video recorded? what was the rationale?

Response: Thank you for your comment. As described in the data collection section of the manuscript, video recordings were specifically used for interviews with participants who had hearing impairments. A team member with a hearing impairment conducted video-recorded interviews for participants with hearing impairment, supported by a sign language

interpreter. The rationale for video recording was to allow the sign language interpreter to voice over the videos, which were then transcribed for analysis. To ensure participant comfort and confidentiality, we followed strict protocols, including obtaining informed consent for the video recording and securely storing the video files. We had added further explanation to the manuscript (see lines 185-193)

Absence of healthcare workers at the facilities? Was this absence or limited?

Response: Thank you for your comment. Participants consistently reported that healthcare workers were often absent from the health facilities, which is a common experience in general healthcare service delivery in rural healthcare facilities. This absence is not merely a matter of limited availability but rather an issue of healthcare workers not being present at the facilities during times when services were needed. This highlights a significant barrier to healthcare access for persons with disabilities in rural settings, where the shortage of healthcare workers is more pronounced compared to urban areas, leading to periods when no healthcare providers are available to attend to patients even after struggling to reach the healthcare facility. Additionally, this clearly demonstrates the rationale for having conducted the study in a rural vs. urban setting.

Comment on marginalisation and poverty

Response: We have been able to clarify how marginalisation, poverty and lack of education can affect the health of persons with disabilities (see line 97-103)

Comment on sentence in lines 114-115

Response: The sentence (lines 114-115) has been corrected and now reads: The country has a high burden of disease dominated by communicable diseases accounting for over 50% of morbidity and mortality, with rural poor communities being greatly affected. (see lines 131-132)

Methods section Did you recruit all ages, sex, etc

Response: Yes, we recruited participants of varied ages and sexes, as represented in Table 1. We have now clarified this in the manuscript by including these categories in the relevant sentence (see lines 160-168).

So, were all the participants approached identified by the above-mentioned groups? Or you were given a list and selected those who were eligible.

Response: Thank you for your query. We initially received a list of potential participants from the mentioned groups (district disability focal person and local disability associations). From this list, we purposively selected those who could represent the various categories of impairment, age and gender for the study. In some cases, we also used snowball sampling to identify additional eligible participants. This approach ensured a diverse and representative sample of persons with disabilities for our study

If this was part of a student's project, you need to make clear above. Otherwise, it doesn't make sense why ethical approval was requested in country where the study was not conducted

Response: Thank you for your comment. To clarify, this study was not part of a student's project but rather an exploratory formative phase of a larger trial sponsored by the London School of Hygiene and Tropical Medicine. Ethical approval was necessary for this formative phase to ensure that all research activities, including those conducted in Uganda, met ethical standards. Even though the study was conducted in Uganda, the sponsorship and oversight by the London School of Hygiene and Tropical Medicine required adherence to their ethical review processes, which included obtaining ethical clearance in the country where the institution is based.

I don't think this is applicable to all persons with disabilities

Response: Thank you for your comment. We have clarified the statement by including some in order to qualify the statement.

Under limited decision making and dependence on carer - The quote is more on dependency not decision-making.

Response: Thank you for the comment. You are correct that the excerpt primarily reflects themes of dependency rather than decision-making. We aimed to include representative excerpts for each theme within the word count constraints but have now added specific examples that illustrate the nuanced experiences related to both dependency and decision-making. To address this, we have included an additional excerpt that more directly represent the aspect of decision-making.

"I am not the one who decides if I need to go to hospital or where to go because of my disability, I just sit there. Also, because am not the one who pays. At the moment, I lack someone to take care of me and to pay for my medical treatment. My daughter who used to do all these things just passed on a few months back".

We hope these additions provide a clearer representation of the issues discussed.

Reviewer: 2

Thank you so much for awarding me the opportunity to review this manuscript. I do believe that the manuscript presents a pertinent issue, especially in the African context. While I do believe that the manuscript is publication worthy, I believe that the following should be addressed before it can be considered for publication:

Abstract:

1. I would suggest adding a sampling strategy in the abstract

Response: Thank you for the suggestion. We have added a brief description of the sampling strategy to the abstract to provide clarity on how participants were selected for the study (See lines 45-48).

2. line 51, correct the typing error. There are two full stops.

Response: Thank you for the observation. We have rectified the punctuation in the sentence

Article summary under strengths and limitations:

Response: Thank you for the observation, we have clarified the section as Strengths and limitations.

3 .Maybe another strength is the use of a persons with disability to collect data from other persons with disabilities, in this case persons who are deaf.

Response: Thank you for this suggestion. We have included the suggestion in the strengths and limitations section (See lines 79-82)

Introduction:

4. Line 93 remove double full stops.

Response: Thank you for the observation. We have rectified the punctuation in the sentence

5. Line 107-108, maybe a brief explanation of why rural Ugandan context is more susceptible to these barriers.

Response: Thank you for your comment. We have expanded on the explanation to provide more context. Rural Ugandan contexts face greater challenges in healthcare access due to

several factors: inequity in the health system, high levels of poverty that limit access to quality healthcare, and a heavy reliance on traditional, alternative, and complementary medicines. These factors collectively exacerbate the difficulties in accessing and utilizing healthcare services in rural areas (see lines 121-124)

Methods:

6. Line 134-135, check the grammar in sentence 135 " This project generating evidence generates evidence to inform......"

Response: Thank you for highlighting the grammatical error. We have revised the sentence to read, "This project generates evidence to inform..."

7. Participants and settings-More clarity is required regarding recruitment. What was done with the lists received from the district focal person?

Response: Thank you for your comment. We initially received a list of potential participants from the mentioned groups (district disability focal person and local disability associations). From this list, we purposively selected those who could represent the various categories of impairment, age and gender for the study. In some cases, we also used snowball sampling to identify additional eligible participants. This approach ensured a diverse and representative sample of persons with disabilities for our study. We have included this in the manuscript (see lines 167-169)

How did you curb form cohesion in situations where the researcher contacted the participants directly and not the participants contacting the researchers?

Response: Thank you for raising this important point. To maintain cohesion and build trust among participants, we implemented several strategies. First, all initial contacts were made respectfully and with thorough explanations of the study's purpose, ensuring participants understood the importance of their involvement. We also provided opportunities for participants to ask questions and voice concerns before agreeing to participate in the study or provide written consent. This was encouraged throughout the time they participated in the study. Furthermore, we conducted interviews in a manner that was culturally sensitive and tailored to individual needs, especially given the varying types of disabilities. By fostering open communication and showing respect for participants' experiences, we aimed to create a comfortable environment that encouraged participation and minimized any potential discomfort. Participants were notified that participation in the study was voluntary; they had a right to withdraw from the study at any time and this would not affect their other rights or privileges. We have added the relevant information to the ethics section of the manuscript (see lines 227-241)

8. Data Collection-More details regarding the persons who conducted the study, their qualification, training on the research methodology. Training of the person who conducted the interviews with deaf participants. etc.

Response: Thank you for the comment. We have included additional details of the persons who conducted the study (see lines 175-180) and conducted the interviews with participants with hearing impairment (see lines 185-193) in the revised manuscript

9. Data Collection line 157-158-Did anyone check the accuracy of the voice overs? If so, who?

Response: Thank you for your question. Yes, the accuracy of the voice-overs was thoroughly checked. The transcripts of the video recordings, which included the sign language interpreter's voice-overs, were reviewed by a bilingual research team member who is fluent in both the local dialects and English. Additionally, the voice-over translations were cross verified with the original video recordings to ensure accuracy. This process was overseen by the first author, who has experience in disability research and qualitative methodologies, to ensure that the translations accurately reflected the participants' responses. We have included this information in the data management section of the revised manuscript (see lines 205-210)

10. Data Collection- How were differences treated in data interpretation?

Response: Thank you for your comment. The differences in data interpretation were managed through a systematic and collaborative approach. During the analysis phase, the researchers involved in coding and interpreting the data discussed their findings in regular team meetings with the entire research group, where researchers with diverse perspectives and expertise contributed to the analysis to ensure that our interpretations were accurate and reliable (see data management section of the revised manuscript).

11. Data management and analysis-Who did the translations from Lusoga and Luganda?

Response: Thank you for the comment. The translations from Lusoga and Luganda were conducted by the trained social scientists (AS, and SS) who were also responsible for conducting the interviews. The researchers are fluent in these local dialects and have extensive experience in qualitative and disability research, ensuring the accuracy and cultural relevance of the translated data. We have included a statement in the methods section (see lines 203-215).

When translations were done, how was it ensured that no meaning was lost?

Response: Thank you for the comment. The recordings were transcribed in the local dialect and later translated to English. The accuracy of the translations was verified by a Sociologist on the research team (AM), who is a native speaker of both local languages. The data management process was overseen by the first author (ASS), who has experience in disability research and qualitative methodologies, to ensure that the translations accurately reflected the participants' responses. We have included a statement in the methods section (see lines 203-215)

12.How was trustworthiness, the equivalent of validity and reliability in qualitative research ensured?

Response: Thank you for raising this important point. To ensure the trustworthiness and rigor of our study, we employed several approaches aimed to uphold the highest standards of rigor and trustworthiness in our research. We have detailed these approaches in the rigor and trustworthiness section of the revised manuscript (see lines 252-280) to provide a clearer account of how we ensured the validity and reliability of our study. For example, we considered maximum variation of the sample by deliberately seeking to include participants with a range of different impairments to capture a broad spectrum of experiences and perspectives. The face-to-face in-depth interviews were majorly conducted in convenient and private participant-suggested locations including the participant's home, community hall, or health facility compound to ensure balance of power during the interviews. The analytical process was conducted independently by two researchers (ASS and SS) who have extensive experience in disability research and qualitative research methodologies. Data from the interview and fieldnotes were triangulated, also to ensure that the results were confirmable. Additionally, the research team had multiple debriefing sessions to discuss emerging themes and ensure a consistent and comprehensive interpretation of the data. At these session meetings, we critically evaluated our biases and assumptions throughout various the study stages. The team members involved in the study have expertise in disability research, participatory community-based research, health systems, and qualitative research methodology.

13. Results- In the argument for the study (introduction section), reference was made to the rural context of Uganda compounding access to healthcare. Even in the discussion similar reference is made using Neil and Penn, 2015. How many of these participants were from rural and how many were from urban areas? This information needs to be mentioned.

Response: Thank you for your comment. The study was conducted exclusively in the rural Luuka district of Uganda, and therefore all participants were from rural areas. Our research

did not include participants from urban settings, so the analysis and conclusions are specifically relevant to the rural context. We acknowledge that referencing the rural context in both the introduction and discussion sections is important for understanding the challenges faced in these settings. The focus on rural areas was chosen deliberately to address the specific barriers to healthcare access that are more pronounced in such environments compared to urban areas. To clarify this in the manuscript, we have revised the methods sections to explicitly state that all participants were from rural Luuka district, Uganda (see lines 157-159)

14. Findings-I would suggest that in the presentation of the results the tenets of the framework are specified and so should the themes and sub-themes for ease of reference. The way in which the results are currently presented requires the reader to deduce this information.

Response: Thank you for your insightful feedback. The results section aligns our findings with the tenets of the theoretical framework used in our study namely Autonomy and awareness; Affordability; Human resources; Health facilities and Assistive technology and Rehabilitation as per Figure 1; defined at the beginning of the results section (see lines 283-284).

15. Findings 205-206-This makes it seem like these are the main methods of health information sharing, however then in lines 213-216, it seems like there are more avenues for information sharing within the healthcare facilities. Please make sure that there are no contradictions.

Response: Thank you for pointing out the potential inconsistency regarding the methods of health information access. To clarify, our manuscript describes two different mechanisms for disseminating health information:

Lines 205-206: focus on the primary methods of health information sharing to the general public that were highlighted by our participants, while Lines 213-216: describe additional avenues for information sharing that were identified within the healthcare facilities that are compounded by accessibility challenges.

16. Findings-The results are interesting but limited to the themes. The sections on the different themes present explanations related to the theme. The themes encompass a lot of elements (sub themes), but one excerpt pertaining to only one aspect of the different elements presented in the one theme. Better to present an excerpt about a sub-theme in a paragraph as opposed to the theme. For example, in the theme of lack of confidence to seek care, there is a lot to unpack, which is bundled as "lack of confidence". Key issues which should be stand-alone sub-themes e.g. stigma should be teased out further, HCP attitudes, service delivery etc.

Response: : Thank you for highlighting this issue. We acknowledge that including participant excerpts is essential for providing clarity and supporting the findings presented in the results section. However, given the expansiveness of qualitative results, we selected representative excerpts that encapsulated the core issues related to each theme. We were unable to include as many quotes as we would have liked. We however, included representative direct participant excerpts that best illustrate the key themes, ensuring that the narrative around the findings is as clear and detailed as possible, reflecting the perspectives shared by participants during the study. We understand that this might limit the depth of some sub-themes, but it was necessary to present the most pertinent information to manage the length of the results section. We have included some additional excerpts from the data to various themes of the study.

17. Discussion- The discussion section is very wanting.

Response: Thank you for your feedback regarding the discussion section. I appreciate your insights and have tried to expound on various aspects of the discussion.

18. References-It is very concerning that dated references, some as far back as 2013 have been used when there is current literature on access to healthcare in Sub Saharan African Countries from 2019- currently. Can this be addressed please

Response: Thank you for your feedback regarding the age of some references used in our manuscript. We understand the importance of incorporating current literature to ensure our research reflects the most recent developments in the field. On the other hand, we also acknowledge the importance of presenting both historical and current perspectives to provide a comprehensive view of the research context. Therefore, in our manuscript, we have included a range of literature that spans both past and present studies, ensuring that all references are relevant and within a 10-year range. This approach was chosen to maintain the timeliness of the data while also drawing on foundational research that provides important context and background.

In response, we have reviewed and updated our references to include more recent studies. This update ensures that our manuscript reflects the latest evidence and perspectives on access to healthcare in Sub-Saharan Africa. While we have included current literature, some foundational references in the introduction section, such as the 2013 review by Soors, Dkhimi, and Criel, provide essential context and background that are relevant to our study's

framework as there are few studies looking at the lack of access to health care for African indigents from a social exclusion perspective similar to what grossly affects persons with disabilities.. This foundational work helps to contextualize our findings within the broader historical and theoretical landscape of healthcare access.

19. Editorial care- I would recommend that this manuscript undergoes editing.

Response: Thank you for your feedback and for recommending additional editing of the manuscript. We have made several revisions to address the concerns raised by the reviewers, focusing on improving clarity, coherence, and the overall quality of the manuscript to ensure we deliver a well-edited manuscript that meets the expectations of the reviewers and the journal.

Thank you very much for considering this research article for publication with BMJ Open, and we look forward to hearing from you. As a result of the suggested revisions, our word count now stands at 6,300.

Sincerely,

Andrew Sentoogo Ssemata.

VERSION 2 - REVIEW

**Reviewer**	**2**
**Name**	**Masuku, Khetsiwe P**
**Affiliation**	**University of the Witwatersrand Johannesburg, Speech Language Pathology**
**Date**	**10-Sep-2024**
**COI**	**I have no competing interests**

The authors have adequately addressed all comments and concerns raised in the previous review.

VERSION 2 - AUTHOR RESPONSE

18th September 2024.

To: Nicole Martin

Research Editor

BMJ Open

Dear Editor,

We are resubmitting a research article titled “Exploring the barriers to healthcare access among persons with disabilities: a qualitative study in rural Luuka district, Uganda.” for consideration for publication in BMJ Open with point-by-point response to the editor’s comments as below:

.

Sincerely,

Andrew Sentoogo Ssemata.
